# Machine learning approach for the prediction of postpartum hemorrhage in vaginal birth

**DOI:** 10.1038/s41598-021-02198-y

**Published:** 2021-11-19

**Authors:** Munetoshi Akazawa, Kazunori Hashimoto, Noda Katsuhiko, Yoshida Kaname

**Affiliations:** 1 Department of Obstetrics and Gynecology, Tokyo Women’s Medical University Medical Center East, Arakawa-ku, Nishiogu 2-1-10, Tokyo, 116-8567 Japan; 2 SIOS Technology Inc., Tokyo, Japan

**Keywords:** Medical research, Mathematics and computing

## Abstract

Postpartum hemorrhage is the leading cause of maternal morbidity. Clinical prediction of postpartum hemorrhage remains challenging, particularly in the case of a vaginal birth. We studied machine learning models to predict postpartum hemorrhage. Women who underwent vaginal birth at the Tokyo Women Medical University East Center between 1995 and 2020 were included. We used 11 clinical variables to predict a postpartum hemorrhage defined as a blood loss of > 1000 mL. We constructed five machine learning models and a deep learning model consisting of neural networks with two layers after applying the ensemble learning of five machine learning classifiers, namely, logistic regression, a support vector machine, random forest, boosting trees, and decision tree. For an evaluation of the performance, we applied the area under the curve of the receiver operating characteristic (AUC), the accuracy, false positive rate (FPR) and false negative rate (FNR). The importance of each variable was evaluated through a comparison of the feature importance calculated using a Boosted tree. A total of 9,894 patients who underwent vaginal birth were enrolled in the study, including 188 cases (1.9%) with blood loss of > 1000 mL. The best learning model predicted postpartum hemorrhage with an AUC of 0.708, an accuracy of 0.686, FPR of 0.312, and FNR of 0.398. The analysis of the importance of the variables showed that pregnant gestation of labor, the maternal weight upon admission of labor, and the maternal weight before pregnancy were considered to be weighted factors. Machine learning model can predict postpartum hemorrhage during vaginal delivery. Further research should be conducted to analyze appropriate variables and prepare big data, such as hundreds of thousands of cases.

## Introduction

Postpartum hemorrhage (PPH) is the leading cause of morbidity in pregnant women worldwide and in Japan. The treatment of PPH has progressed during the last few decades. A variety of compression sutures have been reported^1,2^, the interventional radiology (IVR) has spread and the product of the fibrinogen and blood transfusion strategy has changed^3–5^. However, most of these treatments can only start in tertiary centers. In expected cases such as previa or placenta accreta, we can start the management of PPH using these treatments. However, in an unexpected case, the management of PPH is challenging. Once an unexpected PPH occurs in a community clinic or midwifery home, the time needed for transfer to a tertiary center determines the prognosis of the women.

Although an unexpected massive hemorrhage in a vaginal birth is still a clinical burden, the prediction of PPH remains a challenge. Historically, many risk factors related to PPH have been studied. In a study published in 2013, the strongest independent risk factors for massive blood transfusion included abnormal placentation, placental abruption, severe preeclampsia, and intrauterine fetal demise^6^. In another study using a multivariable analysis published in 2005, significant risk factors were the retained placenta, failure to progress during the second stage of labor, placenta accreta, laceration, instrumental delivery, and large for gestational age newborns^7^. Aside from an abnormal placentation, such as placenta accreta or previa, major risk factors are lacking, and the prediction of PPH is challenging in vaginal deliveries. To decrease the maternal morbidity owing to PPH in a vaginal birth, it is desirable to extract high-risk women for PPH before vaginal delivery.

Recent advances in computer science have driven the development of artificial intelligence (AI). Conventional general programming algorithms produce outputs using the input data and the given rules, whereas AI can produce rules and patterns using the input and output data. The pattern recognition and prediction performance of AI has been demonstrated in multiple realistic tasks. Among a variety of algorithms, deep learning has shown a significant performance and has spread to the medical field.

To save PPH cases worldwide in vaginal birth, the prediction and subsequent stratification of high-risk women is considered essential. A prediction model that integrates a variety of small risk factors and accurately produces the overall risk is required. In this study, we attempted to construct a deep learning model to predict PPH in vaginal birth.

## Methods

### Dataset

All women who underwent vaginal deliveries at the Tokyo Women Medical University East Center between 1995 and 2020 were included. Inclusion criteria for the women were vaginal deliveries with proper women information. Exclusion criteria were cesarean section, miscarriage, death of the baby, and lack of women information. Emergent cesarean section during labor, such as arrest of labor or non-reassuring fetal status (NRFS), was also excluded. The deliveries before 22 weeks and the case of extremely small babies (the weight of neonates under 500 g) were excluded. The study was approved by the institutional review board (IRB) of Tokyo Women's Medical University. Informed consent was waived by the IRB of Tokyo Women's Medical University since this study was retrospective, and the personal information in the data was blinding. The present study was designed and conducted in accordance with the relevant guidelines and regulations of the ethical principles for medical research involving human subjects, as stated by the WMA Declaration of Helsinki.

Each women had 11 variables and one outcome (blood loss). The 11 variables were as follows: (1) age, (2) parity, (3) maternal height, (4) maternal weight before pregnancy, (5) maternal weight upon admission of labor, (6) pregnant gestation of labor, (7) birthweight of baby, (8) sex of baby, (9) fetal position (breech or cephalic delivery), (10) oxytocin use before delivery for induction of labor or uterine inertia, and (11) the model of delivery (spontaneous delivery, vacuum delivery, forceps delivery). Variables 1–7) are continuous numerical data, and variables 8–11) are categorical data. Missing data were processed in two ways: (1) deletion of the case including missing data and (2) replacement of the data with mean values. We analyzed the performance of the models in the two ways. Regarding blood loss, postpartum hemorrhage was defined as blood loss of ≥ 1000 mL within 24 h.

We explained the routine management of vaginal deliveries at our institute. The women received transfusion before the active phase of delivery and one ampoule oxytocin infusion routinely after vaginal birth. If the bleeding continued regardless of the use of one ampoule of oxytocin, more oxytocin or methyelgotamine was added depending on the condition of the women.

### Prediction model

**Figure 1:**
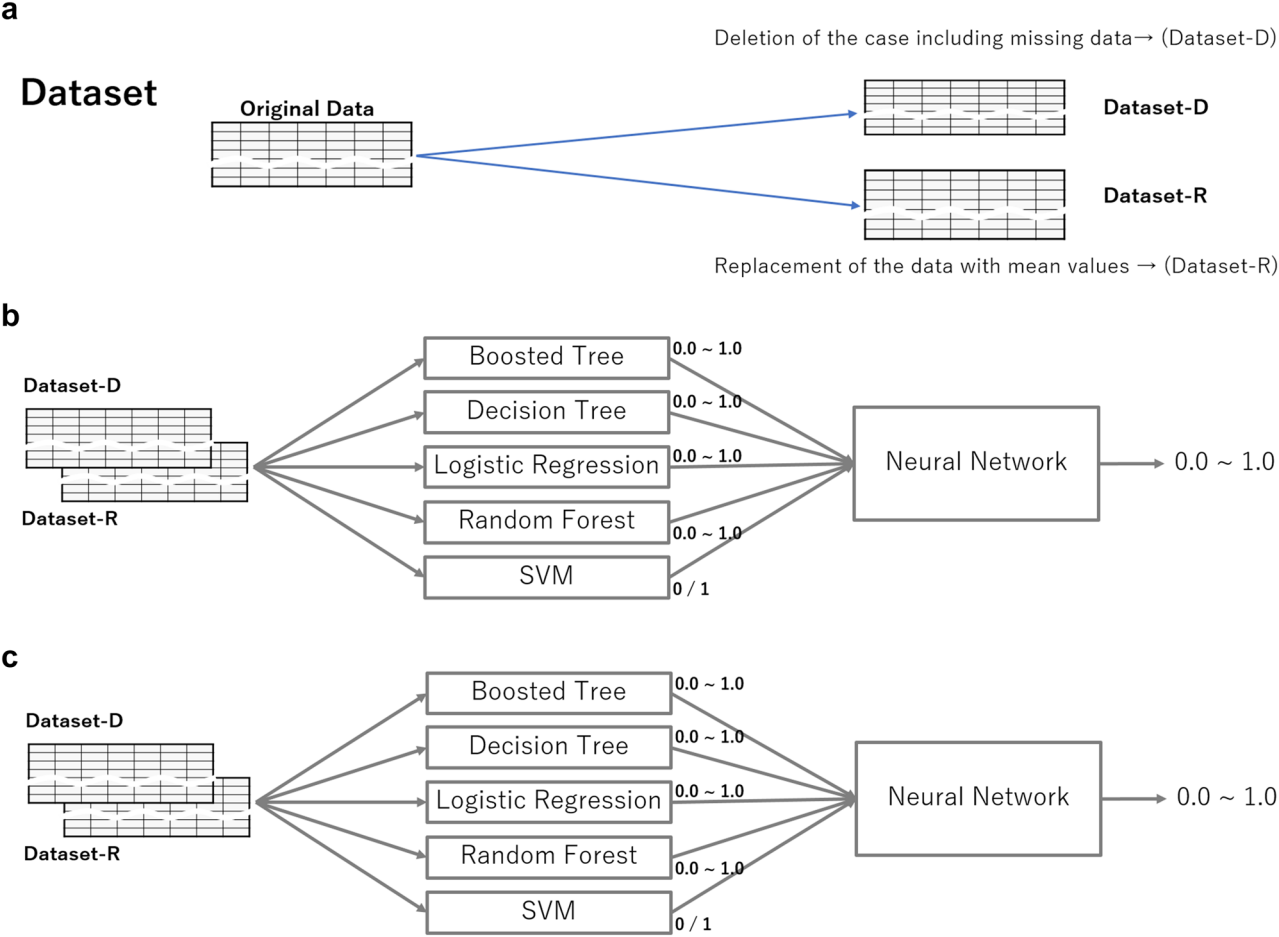
An illustration of the analysis pipeline.

We constructed a deep learning model consisting of neural networks with two layers after the ensemble learning of five machine learning classifiers, including logistic regression, a support vector machine, random forest, boosting trees (XGboost) and decision tree. First, we generated the two dataset from the original data by the processing the missing data in two ways (Fig. 1a). Next, the input data were incorporated into the five machine learning classifiers, producing values using the activation functions (Fig. 1b). Therefore, the values were incorporated into a neural network consisting of two layers for the prediction of PPH (Fig. 1c). We used the L2 normalization for feature normalization for the machine learning models except the tree-based models. We did not perform feature normalization for the neural network. The output of each machine learning models, which was input of the deep learning, was in the range of 0–1. Thus, feature normalization was not considered required for the deep leaning model. We showed the illustration of all the pipeline for the models (Fig. 1a–c).

For validation, we used a k-fold cross-validation as internal validation. The 9,894 cases were randomly assigned to the “training set” (80%) or “test set” (20%) using a random number generator. The rate of PPH groups and non-PPH groups in training set and test set was kept equal to the original dataset. Using the training set, we arranged the parameters of the prediction models and evaluated the performance using the “test set.” The average performance was reported by repeating these five times. For the performance evaluation, we used the AUC of the receiver operating characteristic, that is, the C statistics, as well as the accuracy. We also evaluated false positive rate (FPR) and false negative rate (FNR). The AUC is frequently used as an evaluation value for prediction tasks in data science. The accuracy was calculated as follows: (accuracy) = (correctly predicted as non-PPH cases) + (correctly predicted as PPH cases) / total case. In addition to deep learning, we analyzed the prediction performance of the four machine learning models using similar methods.

First, we conducted a statistical analysis to determine the importance of each variable. Dividing into two groups based on the amount of blood loss (PPH and non-PPH groups), we analyzed the statistical differences between groups. Second, we analyzed the odds ratio of each variable in the univariate analysis. Finally, the feature importance of the variables calculated using a boosted tree was evaluated.

### Statistic technique

A student’s t-test was used to analyze significant differences in the quantitative parameters, and a Fisher’s exact test was used for the qualitative parameters. Statistical significance was set at *p* < 0.05. Statistical analyses were conducted using R statistical software. Machine learning and deep learning was implemented in Python as a programming language.

## Results

### Women characteristics

Of the 9,894 women enrolled in the study, 188 cases (1.9%) had blood loss of > 1000 mL. The characteristics of the women are summarized in Table 1. The median estimated blood loss was 276 mL. The median age was 31 years, and primipara was the most frequent case owing to an increase in the average age of pregnant women in Tokyo. The median pregnancy week for labor was 39 weeks of gestation, and the median birthweight of the baby was 3038 g. Regarding a breech delivery, because we recently chose a cesarean section for breech delivery, the rate of breech deliveries was much smaller (0.5%). Oxytocin use before delivery was conducted in 20% of vaginal deliveries, and vaccum/forceps deliveries were applied in 5% of cases. The higher rate of oxytocin use before delivery seemed to be because our institute is a tertiary prenatal medical center. The number of missing data for each variable are shown in Supplementary Material.

**Table 1:** The characters of all the women.

		All
	Blood loss (mL)	276 (4851–10)
1	Age (year)	31 (45–15)
2	Parity	0 (9–0)
3	Maternal height (cm)	158 (180–131)
4	Maternal weight before pregnancy (kg)	52 (144–31)
5	Maternal weight on admission of labor(kg)	62 (127–29)
6	Pregnant gestation of labor (week)	39 (42–22)
7	Birth weight of baby (g)	3038 (2699–506)
8	Sex of baby	
	Male	51.10%
	Female	48.90%
9	The fetal position	
	Cephalic delivery	99.50%
	Breech delivery	0.53%
10	Oxytocin use before delivery	20.30%
11	Model of delivery	
	Spontaneous delivery	94.80%
	Vacuum delivery	4.10%
	Forceps delivery	1.10%

(1–7) median value ((max-value) − (min-value)), (8–11) the rate.

We divided all cases into PPH groups (> 1000 mL) and non-PPH groups (< 1000 mL), evaluating the statistical difference between them (Table 2). There was a statistical significance for age, height, maternal weight before pregnancy, maternal weight upon admission of labor and birthweight of baby among quantitative variables. For the quantitative variables, although there was no significant difference in the sex of the baby or the breech position, oxytocin use before delivery and forceps/vacuum deliveries showed significant differences. In the univariate analysis, although the significance was shown in the several quantitative variables, the odds ratio of each variable except parity was nearly one. Among these seven quantitative factors, the parity was considered to be slightly important. For qualitative variables, there was also no significant relation between the sex of the baby and the breech position. Similarly, the oxytocin use before delivery and forceps/vacuum deliveries showed a significant difference, which was considered as a predictive variable.

**Table 2:** Evaluation of the statistical difference between PPH groups (> 1000 mL) and non-PPH groups (< 1000 mL).

		PPH	non PPH	*p*-value	OR (95% CI)	*p*-value
	N	9706	188			
	Blood loss (mL)	312	1402			
1	Age (year)	30.9	32.4	< 0.05	1.06 (1.03–1.10)	< 0.05
2	Parity	0.62	0.37	0.21	0.62 (0.49–0.79)	< 0.05
3	Maternal height (cm)	158.6	159.4	< 0.05	1.03 (1.01–1.06)	< 0.05
4	Maternal weight before pregnancy (kg)	53.2	57.2	< 0.05	1.04 (1.02–1.05)	< 0.05
5	Maternal weight on admission of labor(kg)	63.1	67.5	< 0.05	1.04 (1.03–1.06)	< 0.05
6	Pregnant gestation of labor (wk)	38.8	39	0.2	1.06 (0.96–1.17)	0.19
7	Birth weight of baby (g)	3019	3153	< 0.05	1	< 0.05
8	Sex of baby			0.51	0.91 (0.67–1.21)	0.51
	Male	51.50%	48.90%			
	Female	48.50%	51.10%			
9	The fetal position			0.08	3.07 (0.95–9.93)	0.06
	Cephalic delivery	98.40%	99.48%			
	Breech delivery	1.59%	0.52%			
10	Oxytocin use before delivery			< 0.05	1.68 (1.22–2.31)	< 0.05
	oxytocin use	20.10%	29.70%			
	spontaneous delivery	79.90%	90.30%			
11	model of delivery			< 0.05		
	spontaneous delivery	95.14%	84.57%			
	vacuum delivery	0.98%	3.19%		3.63 (1.57–8.41)	< 0.05
	forceps delivery	3.86%	12.23%		3.56 (2.27–5.58)	< 0.05

(1–7) mean value, (8–11) the rate.

*OR* odds ratio, *PPH* postpartum hemorrhage.

### Accuracy of AI models

**Figure 2:**
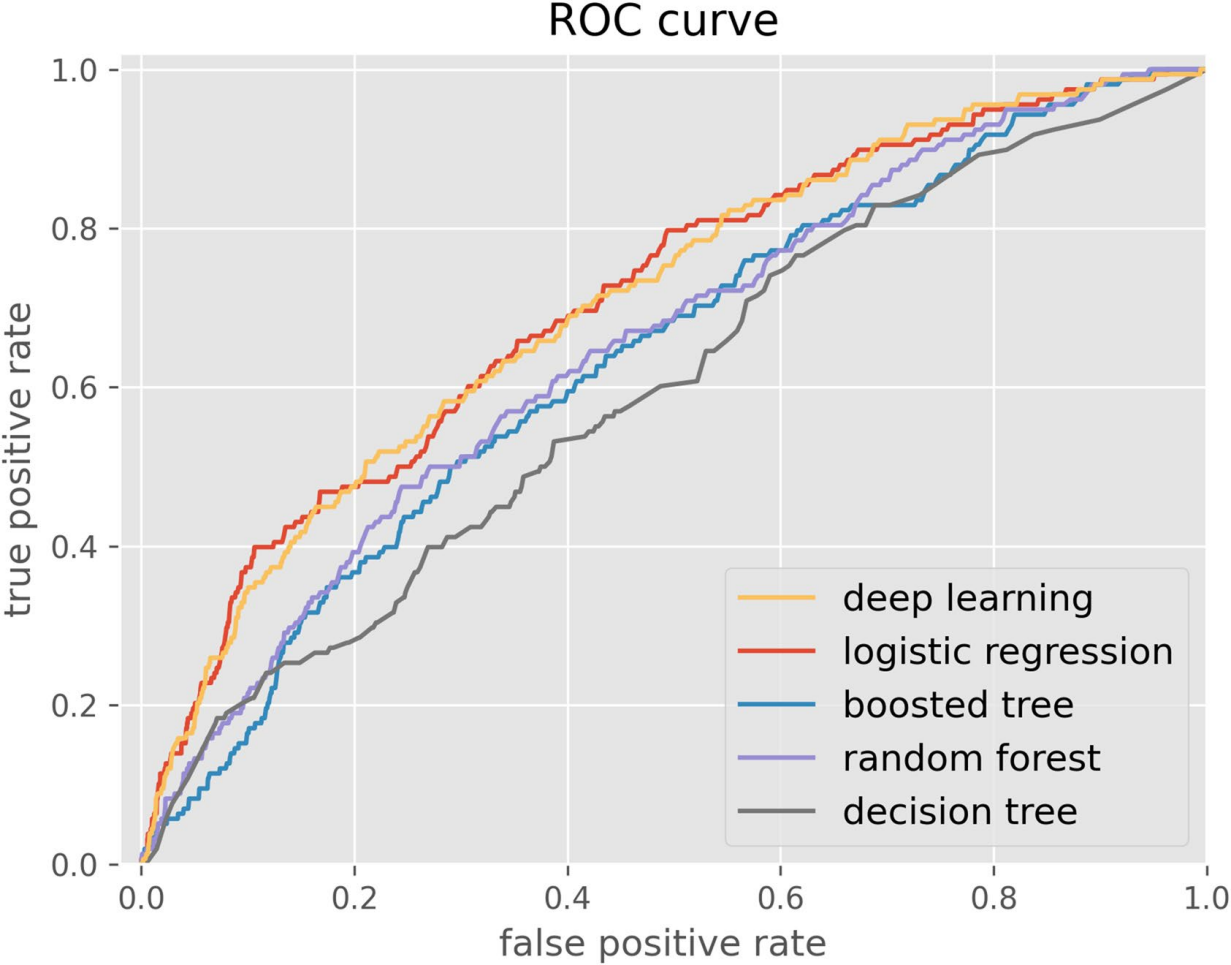
The ROC curves of the deep learning and four machine learning approaches.

The AUC in our deep learning model was 0.706 with the accuracy of 0.681, the FPR of 0.326, and the FNR of 0.379 in deletion of missing data (Table 3). In replacement by mean-value, the AUC was 0.674 and the accuracy was 0.654. Among the four machine learning models, the logistic regression showed the best performance of 0.708 in AUC and 0.686 in accuracy and 0.312 in FPR and 0.398 in FNR, followed by random forest, boosted trees and decision trees. Processing the missing data, we used two methods: (1) deletion of the column of missing data and (2) replacement by the mean values. Between these two methods, by deleting the column of missing data, the AUC showed a better performance between deep learning model and logistic regression. The ROC curves of the deep learning model and four machine learning approaches in deletion of missing data are shown in Fig. 2.

**Table 3:** The performance of deep learning and machine leaning models.

Model	(1) Deletion of missing data				(2) Replacement by mean-value			
	AUC	Accuracy	FPR	FNR	AUC	Accuracy	FPR	FNR
Deep learning	0.706	0.681	0.326	0.379	0.674	0.654	0.351	0.414
Logistic regression	0.708	0.686	0.312	0.398	0.681	0.688	0.311	0.404
Random forest	0.651	0.801	0.186	0.588	0.657	0.791	0.208	0.611
Boosted trees	0.634	0.831	0.158	0.683	0.645	0.821	0.171	0.638
Decision tree	0.596	0.724	0.269	0.601	0.623	0.702	0.292	0.563

*AUC* area under the curve, *FPR* false positive rate, *FNR* false negative rate.

**Figure 3:**
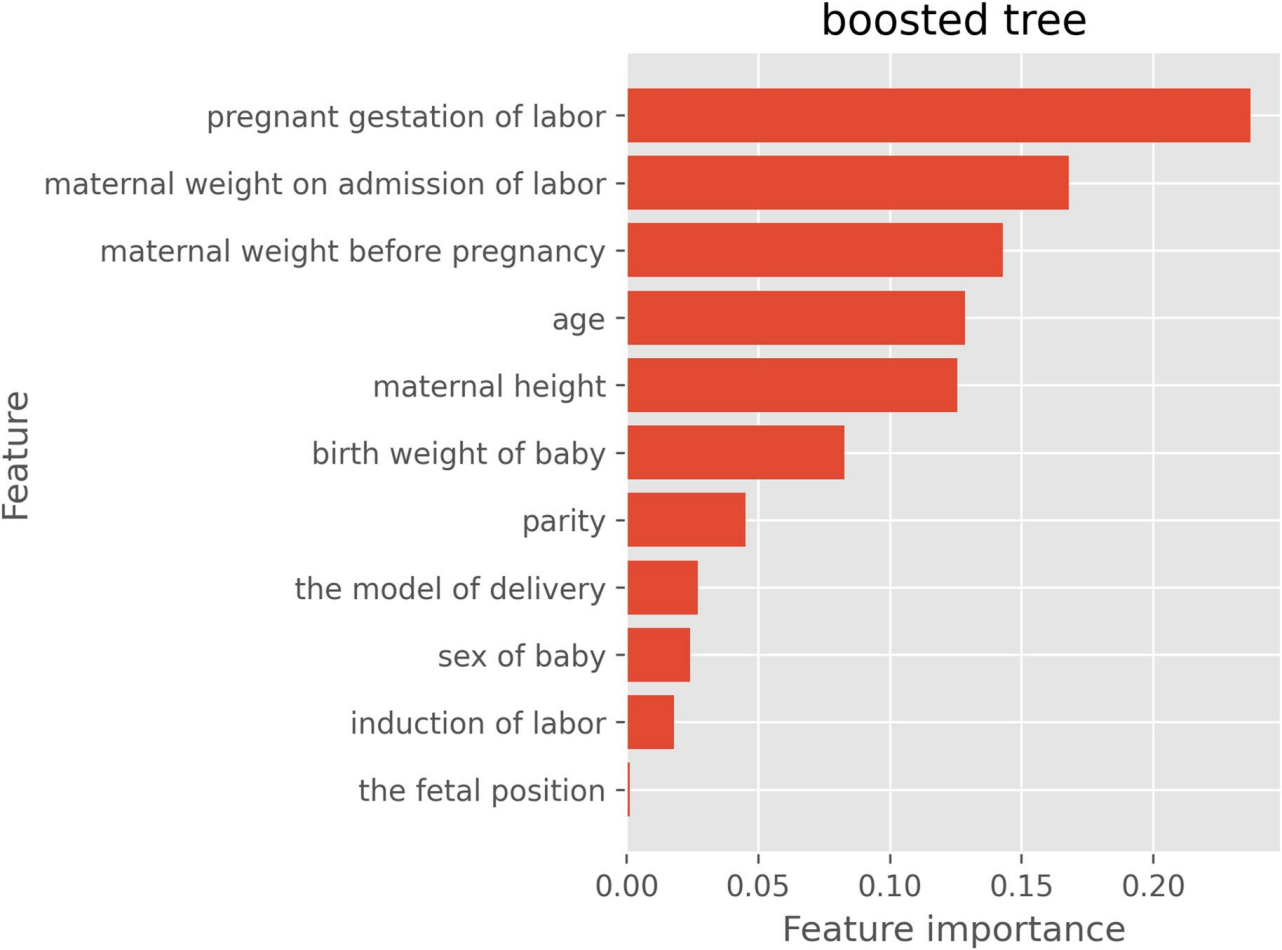
An analysis of the importance of the variables using the boosted tree approach.

An analysis of the importance of the variables using the boosted tree approach showed that pregnant gestation of labor, maternal weight upon admission of labor, and the maternal weight before pregnancy were considered to be mostly weighted (Fig. 3). In a random forest analysis, maternal weight upon admission of labor, pregnant gestation of labor and maternal height were the best predictors.

## Discussion

The prediction of PPH is meaningful in the management of PPH because an accurate prediction can lead to an accurate selection and stratification of high-risk women. Although multiple advances have been made in terms of treatment, little progress has been made in the stratification of women. An unexpected massive hemorrhage in the case of vaginal birth is still a clinical burden. Considering that vaginal births occur worldwide and PPH is the leading cause of maternal morbidity, the selection and stratification of high-risk women is a key to saving maternal lives. A more accurate prediction model and application of a model for daily clinical medicine are desirable.

Historically, many prediction models of PPH have been studied based on logistic regression. In a systematic review published in 2020, 14 prediction models for PPH were developed^8^. The author concluded that three models targeting cesarean section cases have some potential for clinical use among the models. Regarding the prediction mode of PPH in vaginal cases, only three studies were noticed in this review, among which the prediction performance was reported only in one study. Previous studies included in the systematic review have several limitations. As the author commented, the sample size was low, whereas the median size was 788 from 59,468 to 110 cases. The number of variables included was small, whereas the median number of variables was 6, ranging from 15 to 4. In addition, all models used logistic regression for the model construction.

In clinical problems, there are multiple variables influencing the clinical outcome, and the relation between each variable is complex. Linear models, such as traditional logistic regression, might be inappropriate for the statistical and mathematical expression of clinical events. Rather than a linear relation, a non-linear relation with more parameters could be needed for a more accurate prediction model. One solution could be the use of “artificial intelligence (AI)” technology, including machine and deep learning. In particular, deep learning, which contains multiple layers and parameters, can exhibit an excellent performance in realistic predictive tasks.

In 2020, a study using machine learning was published for the prediction of PPH^9^. Of the 152,279 births used in this study, 7279 (4.8%) had a PPH of over 1000 mL. Using 55 risk factors available upon labor admission, machine learning models were constructed, including random forest and extreme gradient boosting. The extreme gradient boosting model achieved the best performance (AUC, 0.93; 95% CI 0.92–0.93), followed by a random forest. This study showed an excellent prediction performance; however, the population of PPH in the study mostly included cesarean cases. The study population included 28% of cesarean section cases, and 91% of PPH cases were cesarean sections.

As a strength of our study, we only targeted vaginal birth. Cesarean section is a major predictor of PPH, and placenta previa is an even stronger predictor. Aside from these factors, the importance of clinical variables is considered weak; thus, no stratification of risk for PPH is performed in clinical situations for vaginal birth. To save unexpected PPH cases occurring in community clinics or midwifery homes, a prediction model for vaginal birth is desired. Second, our dataset consisted of women from a single institute. Blood loss in the vaginal case was difficult to evaluate, compared with cesarean section. Therefore, the method for blood counts for vaginal delivery influenced the estimated blood count among institutes.

As a limitation of our study, first, the size of the dataset was small for the deep learning model. Although we collected 10,000 vaginal cases, the PPH cases only numbered 1000. In medical problems, the positive and negative cases are mostly uneven, which becomes a limitation for learning because the model cannot learn the pattern or features of positive cases from the small number of positive cases. In our study, learning did not progress because of the small number of such cases (PPH cases). Second, we lacked an external validation. We used a cross-validation as the internal validation; however, an external validation was also conducted to evaluate the robustness. Third, we used the neonatal weight, and it could be suggested that the variables cannot be used prior to birth in a realistic manner. However, considering that the size of the baby is an important variable clinically^7^, we included the birthweight of the baby in the model. For clinical application, the estimated birthweight upon evaluation of ultrasound could be used as an alternative to birthweight. Fourth, important variables were lacking. In our study, the importance of each variable calculated using a boosted tree were all weak. Compared with a previous machine learning study^9^, the important variables shown in machine learning models were similar, suggesting that maternal weight and baby weight were the better predictors. However, the values of the feature importance for these variables were small, indicating that these variables were not better predictors. For a better prediction performance, more important variables should be analyzed and incorporated into the prediction models.

In future studies, big data with quality and appropriate variables should be analyzed. The prediction performance will improve with a large dataset because deep learning learns hidden patterns with big data. In reality, the preparation of a large dataset with quality is challenging. When the size of the dataset increases, the missing data should increase, and the number of variables will also decrease. However, the spread of electronic medical records worldwide and database construction for pregnant women in each region or country could benefit the prediction mode through deep learning. With the improvement of the deep learning model with the progress of computer science and the compilation of various medical records as a database, the prediction performance should improve in the future.

## Conclusion

Machine learning and deep learning models can predict postpartum hemorrhage during vaginal delivery. The prediction performance of the model was not good due to the small number of PPH cases and lack of important variables. Further research should be conducted to analyze appropriate variables and prepare big data, such as hundreds of thousands of cases.

## Supplementary Information

Supplementary Information.
